# Risk aversion or adaptation? Public choices in sports participation under climate risks

**DOI:** 10.3389/fpubh.2025.1578845

**Published:** 2025-04-09

**Authors:** Qiuyue Zhang, Long Luo, Xiaobin Guan, Yu Cao, Yili Lin, Yuchen Xiong

**Affiliations:** ^1^College of Economics and Management, Beijing University of Technology, Beijing, China; ^2^Department of Physical Education and Research, Central South University, Hunan, China; ^3^Center for Applied Statistics, Renmin University of China, Beijing, China; ^4^School of Social Research, Renmin University of China, Beijing, China; ^5^School of Political Science and Law, University of Jinan, Shandong, China; ^6^Graduate School of Technology Management, Kyung Hee University, Seoul, Republic of Korea

**Keywords:** climate risk, sports participation, life satisfaction, digital engagement, public health outcomes

## Abstract

**Introduction:**

The increasing frequency and severity of climate risks have significantly impacted public health behaviors, particularly sports participation. Understanding how individuals respond to these environmental shocks is crucial for designing effective health and climate adaptation policies. This study examines the short-term and long-term effects of climate risks on sports participation among middle-aged and young adults, exploring the underlying mechanisms driving these behavioral changes.

**Methods:**

Using data from the 2014 to 2022 China Family Panel Studies (CFPS), this study employs fixed-effects models, two-stage least squares (2SLS) estimation, and a four-stage mediation model to address potential endogeneity and uncover causal relationships. Climate risks are assessed using multiple proxy variables, and robustness checks ensure the reliability of the findings.

**Results:**

In the short term, climate risks significantly reduce the frequency of sports participation. This effect remains consistent across different model specifications and estimation methods. Mechanism analysis reveals that climate risks lower life satisfaction and increase digital engagement, both of which influence individuals' physical and mental health. While climate risks initially discourage sports participation, long-term adaptation occurs through digital engagement and indoor exercise, leading to improved health outcomes. Heterogeneity analysis indicates that the negative short-term effects are more pronounced in urban and western regions, with rural and western areas experiencing no significant long-term positive effects.

**Discussion:**

This study highlights both the inhibitive short-term effects and adaptive long-term responses to climate risks in sports participation. The findings provide insights into how individuals adjust their health-related behaviors under environmental stress and offer policy recommendations to promote climate adaptation and public health through targeted interventions.

## 1 Introduction

Climate change has become a critical and far-reaching scientific issue globally (1). The Intergovernmental Panel on Climate Change (IPCC) reports have clearly indicated a significant and persistent increase in global average temperatures. At the same time, the frequency and intensity of extreme weather events have also increased significantly (2). In recent years, this trend has been evident across several regions: Europe and North America have experienced frequent heatwaves (3), Asia has faced severe flooding and rainfall disasters (4), and the Americas have struggled with intensifying droughts (5). These extreme weather events not only highlight the widespread impact of climate change on Earth's ecosystems, but also pose significant and long-term challenges to human society, including the environment, economic development, and public health (6–8).

Against the backdrop of climate change, sports activities face increasingly severe challenges (9). China has placed a strong emphasis on addressing climate change and its impact on public life. As part of the broader Healthy China initiative, the country has actively promoted mass fitness and is pushing for the development of the sports sector in a green and sustainable direction. Since the implementation of the “National Fitness Program Outline,” public enthusiasm for participating in physical activities has remained high, and sports have become an essential way to improve the physical health and quality of life of the population. However, the risks posed by climate change to physical exercise are becoming increasingly complex and multidimensional (10). As a result, the safety, convenience, and sustainability of sports activities are being compromised by climate change, leading to increased risks and costs (11). In this context, understanding the interplay between climate change and physical exercise has become an urgent necessity for safeguarding public sports rights, and promoting the healthy development of the sports sector.

Although extensive research has been conducted in the academic community on the causes of climate change, its mechanisms of ecosystem disruption, and its impacts on sectors such as the economy and agriculture (12–14), there remains a gap in the study of the relationship between climate change and physical exercise. Existing literature primarily focuses on the impacts of climate change on agriculture (15), the economy (16), and public health (17), with limited empirical analysis on short-term and long-term sports participation behavior. In particular, there is a lack of research on how climate risks affect individuals' decisions to engage in sports, participation frequency, and the development of long-term exercise habits.

Individual sports participation is not only a health behavior choice but also an adaptive decision influenced by multiple factors (18). It is shaped not only by “hard” factors such as economic conditions and policy support but also by “soft” factors like risk perception and adaptation preferences (19). The formation of risk perception and adaptation preferences is closely related to the individual's natural environment and socio-cultural background (20). Ignoring the role of long-term climate exposure, technological development, and other factors in shaping individual behavior may lead to biased understandings of sports participation (21). Furthermore, existing research often overlooks the specific impact of climate risks on different social groups, particularly the middle-aged and young populations, failing to explore the complex interactions between climate risks and individual adaptive behaviors. Studying the adaptive behaviors of various groups in response to climate risks can deepen our comprehensive understanding of the impacts of climate change and provide valuable insights for effective policies and interventions. Therefore, this study adopts a perspective of social adaptability, focusing on the sports participation behaviors of middle-aged and young people in China. The aim is to explore how individuals adjust their behaviors in response to climate risks. The findings will contribute to a deeper understanding of the determinants of sports participation behaviors.

Based on this, the climate risks individuals experience in different regions may influence their preferences and participation decisions in physical activities by shaping their risk perception (22). However, the ways in which climate risks shape social adaptation and how these patterns affect short-term adjustments and long-term habit formation in individual fitness behavior remain insufficiently explored and empirically tested. Therefore, this study explores the following core questions: (1) How does climate risk influence individual sports participation, and through what mechanisms? (2) Do climate risk-driven social adaptation patterns, such as the proliferation of digital lifestyles, mediate the long-term impact of climate risk on sports participation? (3) How do regional differences in climate risk contribute to the heterogeneity of sports participation behavior?

To address the above questions, we use provincial-level climate risk data from China to examine both the short-term and long-term impacts of climate risk on the exercise frequency of middle-aged and young adults. The results indicate that climate risk significantly reduces the frequency of sports activities among middle-aged and young adults in the short term. To verify the robustness of this conclusion, we conducted additional robustness tests, including replacing key explanatory and dependent variables, altering model specifications, and adjusting estimation methods. The results consistently support the conclusion across all robustness tests. Furthermore, to address the endogeneity issue between climate risk and fitness behavior, we use the average climate risk levels from other provinces in the same year as an instrumental variable, and apply the 2SLS method for regression, with the results remaining robust.

Further mechanism analysis shows that climate risks reduce life satisfaction, which in turn leads to a decrease in the frequency of physical activity among middle-aged and young individuals. However, the rise in climate risk also accelerates the adoption of a digital lifestyle, which, in turn, promotes an increase in exercise frequency in the long term. Both mechanisms are supported, indicating that climate risk has a short-term inhibitory effect and a long-term adaptive adjustment effect on fitness behavior. Heterogeneity analysis shows that the short-term negative effect of climate risk is more pronounced in rural and western regions, with no significant positive long-term effect. This suggests regional differences in individuals' adaptive adjustments to climate risk.

The potential contributions of this study are as follows: First, in the context of increasing climate risk, it examines the impact of climate risk on the fitness behavior of middle-aged and young adults, expanding research on environmental shocks and health behaviors, and providing empirical evidence from China for understanding individuals' adaptive strategies to environmental risks. Second, it enriches research on the drivers of fitness behavior. While existing studies mainly focus on socioeconomic conditions, health perceptions, and policy environments, this study emphasizes external environmental factors and systematically explores the short-term and long-term effects of climate risk on fitness behavior. Third, it deepens understanding of individual behavioral adjustment mechanisms under environmental shocks. This study finds that climate risk shapes the dynamic patterns of fitness behavior by influencing life satisfaction and promoting the adoption of a digital lifestyle, further enriching theoretical perspectives on individuals' responses to environmental challenges, and advancing our understanding of the interplay between climate change and human lifestyles.

## 2 The literature review and research hypotheses

### 2.1 Literature review

Climate risk refers to uncertainties arising from climate factors such as extreme weather, natural disasters, global warming, and the societal transition toward sustainability (23). It encompasses both physical risks caused by meteorological disasters and transition risks associated with the green transformation process (24, 25). Physical risks primarily manifest as acute shocks from extreme weather events and long-term pressures resulting from changes in climate patterns. These include asset destruction caused by meteorological disasters like hurricanes, floods, and droughts, as well as long-term risks such as disruptions in agricultural production cycles and the depreciation of coastal assets due to the greenhouse effect (26). Transition risks arise from industrial restructuring within the carbon neutrality policy framework, including increased compliance costs due to carbon pricing mechanisms, stranded asset risks from the substitution of clean technologies (27), and challenges faced by traditional sports equipment manufacturers due to the adoption of clean technologies amidst tightening environmental regulations (28).

Climate risks impact the sports sector on multiple levels, including macro, meso, and micro levels. At the macro level, the effects primarily involve infrastructure development and maintenance, changes in market demand and consumption patterns, and supply chain disruptions. First, extreme weather events such as heavy rain, hurricanes, and floods can damage sports venues and outdoor facilities, leading to facility destruction or collapse. Repairing or reconstructing these facilities incurs significant costs and leads to prolonged closures, disrupting normal sports activities and events (1, 29). Second, climate factors such as global warming and changes in precipitation patterns influence people's preferences for sports. Global warming, for example, significantly impacts the skiing market (30–32). Moreover, excessively hot or cold weather can reduce people's willingness to engage in outdoor activities, shifting participation toward indoor sports (33), thereby altering market demand and prompting a reallocation of resources within the sports industry. Finally, climate risks affect the supply and price of raw materials for sports products, such as wood (34) and cotton (35), which are sensitive to climate conditions. Extreme temperatures can lead to crop failures or quality deterioration (36), resulting in raw material shortages and delays in sports product production (37).

At the meso level, the impact of climate risks is primarily seen in event organization and commercial operations. Events may face more frequent climate-related risks (38), such as extreme weather leading to delays or cancellations, which disrupt the smooth execution of the event. The level of investment by event sponsors is typically inversely related to climate risks, as the unpredictability of extreme weather events may cause fluctuations in advertising effectiveness, affecting sponsors' decisions (39). Furthermore, the viewing experience under harsh weather conditions significantly reduces audience engagement and satisfaction (40, 41), which can affect their willingness to attend future events and potentially decrease the commercial value of the event (42).

At the micro level, the impact of climate risks primarily affects physical health, willingness to exercise, and exercise habits. Extreme weather increases physical strain, with high temperatures raising the risk of heatstroke (43), and cold weather potentially causing injuries (44). Long-term exposure to smog may also damage the respiratory system, increasing disease risk (45). Additionally, adverse weather often reduces people's willingness to exercise. Extreme conditions not only induce inertia but also exacerbate psychological fatigue, affecting motivation (46). Reduced sunlight or rainy weather can also affect mood, influencing long-term exercise plans (33). Finally, as the climate changes, the choice of exercise venues is shifting, with more people opting for indoor facilities to avoid weather disruptions (46). In economically developed regions, climate risks have also driven the digital transformation of sports, facilitating remote exercise unaffected by weather (47, 48).

Exercise refers to a subcategory of physical activity that is planned, organized, repetitive, and beneficial for maintaining or developing physical health (49). The benefits of exercise are widely recognized, and existing research categorizes the factors influencing participation in physical activity into internal and external factors (18). Regarding internal factors, age is a primary consideration, with some studies indicating that exercise participation decreases with age among adults (50, 51). Gender differences also play a significant role, as men and women are motivated by different factors to engage in physical activity (52, 53). Furthermore, for individuals with poor health, even though exercise can help improve their condition, physical limitations may affect their participation in physical activity (54). Finally, some studies suggest that cultivating self-efficacy is an effective way to motivate people to engage in exercise (55).

From the perspective of external factors, several studies have examined the impact of urban environments (56), institutional support (57), and economic costs (58) on exercise participation. First, in terms of the environment, multi-purpose urban designs play a crucial role in promoting physical activity related to transportation and recreation (59). A supportive exercise atmosphere, such as social and peer support, also facilitates engagement in and adherence to exercise. Second, from an institutional perspective, national policies, and systems can broaden the scope of exercise participation (60, 61). Finally, from an economic perspective, at the national level, compared to developed countries, adolescents in developing countries are in the early stages of understanding physical activity, and exhibit lower levels of physical exercise than their counterparts in developed countries (62). At the regional level, individuals living in wealthier areas have greater access to moderate-to-high-intensity exercise facilities compared to those in poorer areas (63, 64). At the individual level, health inequalities arising from income disparities remain prevalent (65–67).

Climate risks manifest differently across the globe, prompting countries to adapt their sports practices accordingly (68). In Europe and North America, digital transformation and improved infrastructure have mitigated the negative impacts of climate risks on sports participation (69). Furthermore, research shows that the rise of wearable smart devices not only helps athletes monitor their performance data but also provides exercise recommendations based on weather warnings, assisting people in maintaining their exercise routines during extreme weather conditions (70). In contrast, developing countries face greater challenges (71), particularly in regions with weak infrastructure, and frequent climate risks, where sports participation is more heavily restricted (72). This has led to increased attention in these areas toward sports infrastructure and policies to ensure public access to physical activities. Overall, the impact of climate change on the sports sector is multifaceted, encompassing not only the direct effects of weather on health but also the behavioral adjustments people make in response to climate change (73). Although digital transformation, as an innovative approach to addressing climate risks, has been widely applied across various sectors (74), empirical research on its role in promoting sports participation remains limited (23). Additionally, digital development in developing countries and regions is still in its early stages. How to leverage technological means to reduce the constraints of climate risks on sports activities remains a key direction for future research.

### 2.2 Research hypotheses

Frequent extreme weather events, such as heavy rainfall, high temperatures, and severe cold, not only disrupt daily travel but also affect outdoor exercise routines (75). These climatic anomalies directly impact individuals' exercise plans, leading to restrictions or even cancellations of normally regular activities. This uncertainty and disruption often reduce life satisfaction. According to the theory of subjective wellbeing (76), life satisfaction is an overall assessment of quality of life. The negative effects of climate risks on life satisfaction can undermine subjective wellbeing, influencing behavior choices, particularly physical exercise. Additionally, air quality deterioration, haze, and environmental pollution caused by climate change present greater challenges for physical activity among young and middle-aged individuals. For instance, slippery surfaces after rain or snow (77), along with increased air pollution, may raise respiratory disease incidence and exacerbate symptoms in individuals with cardiovascular conditions (78). Therefore, extreme weather and climate risks directly impact physical exercise among young and middle-aged individuals, potentially diminishing their enthusiasm for physical activity (79). Based on this context, the following hypothesis is proposed:

**Hypothesis 1**: Climate risks significantly reduce life satisfaction among young and middle-aged groups, thereby suppressing the frequency and intensity of their physical exercise.

The theory of adaptive behavior emphasizes that individuals or groups adopt strategies to adjust to environmental changes and challenges. Specifically, adaptive behavior refers to actions and strategies individuals or groups use to cope with environmental changes and uncertainties (80). Specifically, adaptive behavior refers to actions and strategies individuals or groups use to cope with environmental changes and uncertainties. This adaptation need has accelerated the development of digital lifestyles (81), particularly in the fitness sector, where practices like online fitness courses, virtual trainers, and smart fitness equipment have emerged. With digital technology, young and middle-aged individuals can mitigate the impact of extreme weather, maintaining their health management and exercise routines (47). The popularity of online fitness courses removes time and space limitations, allowing people to exercise at home or in the office, regardless of weather or busy schedules, overcoming external constraints on exercise (48). At the same time, the rapid development of smart fitness equipment offers real-time data monitoring, helping individuals with more scientifically structured training (82, 83). These devices adjust exercise plans based on feedback data and provide personalized fitness advice, enhancing both enjoyment, and effectiveness. As climate risks rise, more young and middle-aged individuals are opting for digital fitness methods, unaffected by weather. This trend has led to the widespread adoption of digital lifestyles and increased participation in physical exercise. However, according to the digital divide theory, the ability to use internet technology, unequal social resources, and differences in education levels significantly affect an individual's adoption of digital fitness methods (84). That is, groups with higher internet usage awareness are more likely to engage in physical exercise through digital technology, while those with lower internet awareness may continue to be affected by climate risks due to their inability to access such resources (85). Therefore, while digital fitness methods can effectively mitigate the impact of climate risks, their effectiveness is influenced by disparities in internet usage awareness (86). Based on this, the second hypothesis of this study is proposed:

**Hypothesis 2**: Climate risks, by promoting the widespread adoption of digital lifestyles, can contribute to increased physical activity participation among middle-aged and young adults in the long term; however, this effect is influenced by disparities in internet usage awareness.

**Figure 1:**
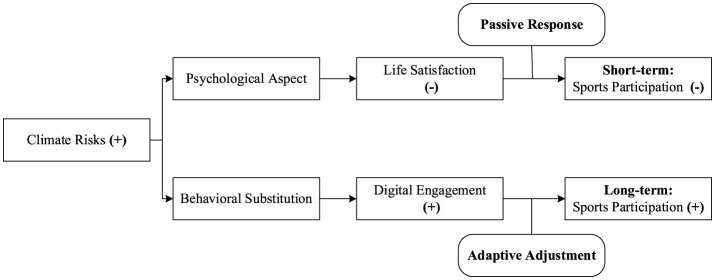
Mediation path diagram.

The logical relationships of the hypotheses in this study are shown in the mediation path diagram in Figure 1.

## 3 Data and descriptive statistics

### 3.1 Data sources and sample construction

The microdata for this study is from the China Family Panel Studies (CFPS), based on systematic household surveys conducted in 2014, 2016, 2018, 2020, and 2022. After filtering the CFPS samples to exclude adolescents and the older adult, the focus was on young and middle-aged individuals aged 16 to 55. After removing missing and invalid data, 23,957 valid samples were retained. Climate-related data were obtained from daily observations by the China Meteorological Station. Other provincial-level data were sourced from the National Bureau of Statistics. To mitigate the impact of outliers, all continuous variables were winsorized at the 1st and 99th percentiles.

### 3.2 Variable selection

#### 3.2.1 Independent variable: regional climate risk

Based on the Climate Physical Risk Index constructed by Guo et al. (87), we use the CPRI to assess climate risks across China's provinces. The construction of this index involves the following steps: first, historical meteorological data (1973–1992) were used to determine standard threshold values for four types of extreme climate events. These events encompass key climate variables such as temperature (LTD, HTD), precipitation (ERD), and humidity (EDD), which comprehensively reflect the potential impact of climate anomalies on ecological and socio-economic systems (88). The number of extreme cold days (LTD) measures the frequency of cold wave events, which are critical for infrastructure and agricultural production safety; the number of extreme hot days (HTD) reflects the intensity of heatwaves, directly impacting public health and energy demand; the number of extreme rainfall days (ERD) characterizes the severity of heavy rainfall, closely linked to flooding and residents' daily lives; and the number of extreme drought days (EDD) gauges the impact of prolonged low humidity on water resources, ecosystems, and living habits. These indicators not only capture the extremity of individual climate events but also characterize the diversity of climate risks from different dimensions, ensuring a comprehensive assessment of the vast, and climatically diverse regions of China.

Using daily observation data from Chinese meteorological stations provided by the National Oceanic and Atmospheric Administration (NOAA), we calculate the number of extreme climate event days at each station. The data is then mapped to the corresponding provinces and prefecture-level cities based on the geographic coordinates of the stations, and regional averages are calculated. To account for regional climate differences and improve comparability, the number of extreme event days for each region is standardized using a min-max scaling method, adjusting the values to a range between 0 and 100. Finally, by calculating the equally weighted average of the four standardized sub-indices (LTD, HTD, ERD, and EDD), we obtain the Climate Physical Risk Index (CPRI) for each region. This index considers various types of extreme climate events, allowing it to reflect the specific risks of individual provinces while enabling horizontal comparison across the country, thereby effectively measuring the diverse climate risk levels in China.

#### 3.2.2 Dependent variable: fitness participation

This study treats sports participation as the dependent variable and uses exercise frequency as an indicator of sports participation among the middle-aged and young population, following Huang et al. (89). Specifically, the CFPS survey's method of asking about exercise frequency has varied across years (continuous values prior to 2018 and categorical values from 2020 onward). We recognize that participants may be influenced by social desirability bias (i.e., responding in a way that aligns with societal expectations) and recall bias (i.e., potentially overlooking or overestimating exercise duration during the recall process). To mitigate the impact of these biases, we applied several data adjustments. In this study, exercise frequency was reclassified into five discrete categories, with higher levels indicating more frequent exercise, in order to reduce potential extreme values in individual reports. At the same time, for the measurement of exercise duration, the study directly used the exercise duration variable from the CFPS (the number of minutes spent exercising). To improve the data distribution and reduce the impact of extreme values, 1 was added to the value, and the logarithm was taken.

#### 3.2.3 Control variables

To ensure the validity and reliability of the research findings, this study selected the following control variables: age, marital status, employment status, health status, education level, income level, local government expenditure on culture, sports, and media, urbanization level, per capita urban road area, and greening coverage rate in built-up areas. The purpose of selecting these variables is to exclude external factors that may interfere with fitness behavior. Specifically, individual characteristics such as age, marital status, employment status, health status, and education level have been widely proven to have a direct impact on exercise frequency and methods (90, 91). In terms of economic conditions, income level and local government expenditure can reflect the level of support for exercise in a region (62). In addition, urban levelization and levels environmental factors (such as road area and greening coverage and rate) may have varying impacts on exercise environmental behavior due to regional differences (92, 93). Additionally, urbanization and environmental factors—such as road area and greening coverage—may have varying effects on fitness behavior due to regional differences (92–94). Therefore, controlling for these variables helps reduce the influence of external factors and improves the accuracy of the model's estimation results. The specific variable definitions are provided in Table 1.

**Table 1:** Variable definitions.

**Variable**	**Definition**
ef	Frequency of exercise, ranging from 0 (never exercise) to 5 (high frequency).
CPRI	Climate Physical Risk Index
age	Respondent's age
marital	Marital status: 1 = single, 2 = married or cohabiting, 3 = divorced or widowed.
employ	Employment status: 0 = unemployed, 1 = employed, 2 = out of the labor market.
lnincome	Natural logarithm of total income at the end of the year
health	Health status: 1 = very unhealthy, 2 = somewhat unhealthy, 3 = average, 4 = somewhat healthy, 5 = very healthy.
edu	Education level: 1 = illiterate, 2 = primary school, 3 = middle school, 4 = high school, 5 = associate degree, 6 = bachelor's degree, 7 = master's degree, 8 = doctoral degree.
lnLGCSM_Exp	Logarithm of local government expenditure on culture, sports, and media.
lnUrbanRoad_PerCapita	Logarithm of per capita urban road area.

### 3.3 Model construction

Building on existing research on the factors influencing sports participation (95, 96), this study further explores the impact of macro climate risks on individual sports participation. Specifically, the following model is used for regression analysis:


(1) $$\begin{array}{l} ef_{i,t}={{\beta_{0}+\beta }_{1}CPRI}_{i,t}{{+\beta }_{2}Z}_{i,t}+\delta_{t}+\varphi_{i}+\varepsilon_{i,t} \end{array}$$

Let *i* represent the individual and *t* represent the year. CPRI denotes the climate risk level in the individual's region, and *Z* is the vector of control variables that may affect the individual's sports participation, as defined in Table 1. This model employs fixed-effects (FE) estimation, including individual fixed effects, year fixed effects δ_*t*_, and provincial fixed effects φ_*i*_, to control for unobservable heterogeneity across individuals, years, and provinces.

### 3.4 Descriptive statistical analysis

Table 2 provides descriptive statistics for the explanatory variables, dependent variable, and control variables. The sample includes 23,957 observations from 2014 to 2022. As shown in Table 2, the average frequency of physical exercise is 1.045, with a standard deviation of 1.549, a minimum value of 0, and a maximum value of 4, indicating generally low exercise frequency with considerable variation. The average value of the CPRI is 43.569, with a standard deviation of 7.364, a minimum of 31.215, and a maximum of 68.579, reflecting varying levels of exposure to climate risk. Regarding personal characteristics, the average age of respondents is 40.353 years, with most being married or cohabiting and employed. There is significant income variation, and the majority report their health status as “average” or “good.” Most respondents have completed middle school or high school. Additionally, the average local government spending on culture, sports, and media, as well as per capita urban road area, are 4.529 and 2.642, respectively, suggesting considerable differences in infrastructure and cultural sports expenditures across the regions in the sample.

**Table 2:** Descriptive statistics.

**Variable**	***N***	**Mean**	**SD**	**Min**	**Max**	**Median**
ef	23,957	1.0449	1.5490	0.0000	4.0000	0.0000
CPRI	23,957	43.5685	7.3640	31.2152	68.5786	43.5970
age	23,957	40.3531	8.7824	24.0000	55.0000	40.0000
marital	23,957	1.8336	0.4366	1.0000	3.0000	2.0000
employ	23,957	1.0834	0.3342	0.0000	2.0000	1.0000
lnincome	23,957	6.0355	5.0022	0.0000	11.7193	9.2104
health	23,957	3.3756	1.1142	1.0000	5.0000	3.0000
edu	23,957	3.1864	1.3943	1.0000	6.0000	3.0000
lnLGCSM_Exp	23,957	4.5285	0.4433	3.8206	5.7741	4.4914
lnUrbanRoad_PerCapita	23,957	2.6417	0.3495	1.4134	3.2492	2.6218
lnLGCSM_Exp	23,957	38.8040	3.6768	30.8000	48.4000	39.3000

**Figure 2:**
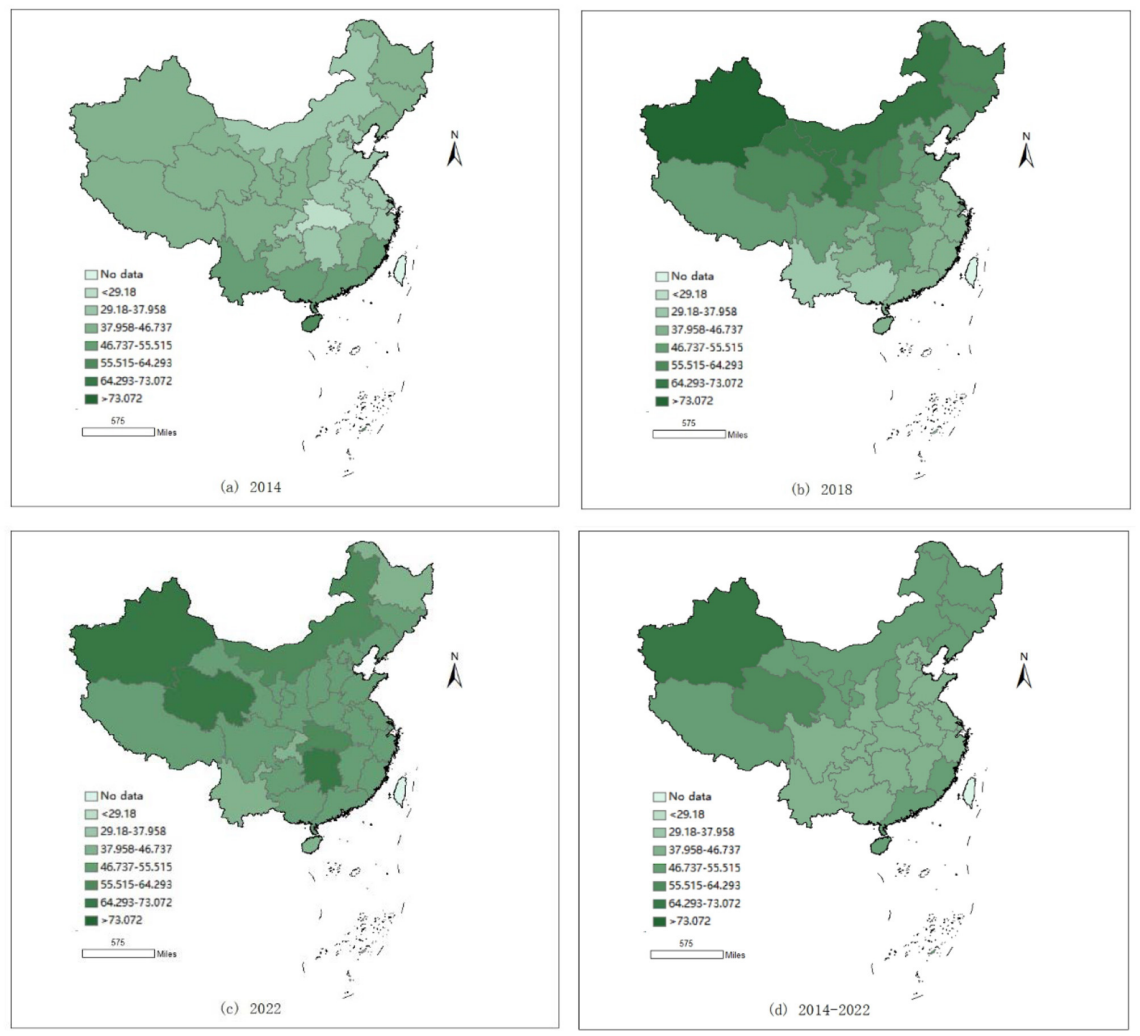
2014–2022 climate physical risk index map.

To more clearly illustrate the spatiotemporal evolution of climate risks across Chinese provinces and cities, this study applies the natural breaks method to categorize climate risk levels from 2014 to 2022 into seven categories. Additionally, using ArcGIS software, spatial distribution maps of regional economic resilience levels for China's 31 provinces, autonomous regions, and municipalities for the years 2014, 2018, 2022, and the 2014–2022 average are generated (see Figure 2).

Figure 2 shows that climate risk levels across Chinese provinces generally follow a pattern of higher risks in the west, lower risks in the east, higher risks in the north, and lower risks in the south. The climate risk levels are relatively lower in the central and eastern regions, while the northwest and northeast regions are notably higher than other areas, displaying clear spatial differentiation. In 2014, the overall climate risk in China's provinces was relatively low, with only a few provinces in the southern border regions exhibiting higher levels. This indicates that, at the time, China's overall climate risk index was low, and the climate environment was relatively favorable; by 2018, a clear reversal occurred. Climate risk levels in the northwest and northeast regions showed a significant upward trend, becoming notably higher than in other parts of the country. Some provinces in the central and eastern regions also exhibited an increasing trend, while climate risk levels in the southern border regions declined significantly. This suggests that the southern regions experienced more climate risks, leading to stronger awareness of climate change and earlier successes in climate environment management. In 2022, climate risk levels in the western regions remained high, while those in the northeast showed a declining trend. Most provinces in the central and eastern regions exhibited an increasing trend, indicating that some accumulated production and construction capacity had been fully released, subsequently impacting the climate environment. From the perspective of the 2014–2022 average climate risk levels, there is an overall pattern of higher risks in the northwest and lower risks in the southeast. The northwest, due to harsh natural conditions, is more susceptible to climate risks, while the southeast benefits from a more favorable natural environment and greater attention to environmental issues by both residents and the government. As a result, effective management measures have led to lower overall climate risks in the region.

## 4 Empirical results

### 4.1 Baseline results

Table 3 presents the baseline regression results for the impact of climate risk on sports participation. Columns 1 through 4 display panel fixed-effect regression results with different sets of information included. The regressions control for individual, provincial, and year fixed effects to mitigate potential biases from sample heterogeneity and time trends. The results show that the coefficient for the CPRI is negative and statistically significant at the 1% or 5% level across all models (the coefficients in columns 1 through 4 are −0.0120, −0.0120, −0.0118, and −0.0100, respectively). This indicates that an increase in climate risk significantly reduces sports participation among middle-aged and young individuals. Specifically, for every one-unit increase in the Climate Risk Index, sports participation decreases by ~0.0100 to 0.0120 units. This result is robust across different model specifications, suggesting that climate risk universally has a negative impact on fitness behavior, with climate change potentially discouraging individuals from engaging in physical activity.

**Table 3:** Baseline regressions.

**Variables**	**(1)**	**(2)**	**(3)**	**(4)**
	**ef**	**ef**	**ef**	**ef**
CPRI	−0.0120^***^	−0.0120^***^	−0.0118^***^	−0.0100^**^
	(−3.01)	(−2.99)	(−2.89)	(−2.38)
Age		0.0005	0.0003	0.0036
		(0.01)	(0.00)	(0.05)
**Marital status (with single as the reference category)**				
2.marital		−0.3035^***^	−0.3034^***^	−0.3161^***^
		(−2.74)	(−2.74)	(−2.85)
3.marital		0.0796	0.0800	0.0482
		(0.36)	(0.36)	(0.22)
**Employment status (With unemployed as the**				
**reference category)**				
1.employ		−0.2894^**^	−0.2897^**^	−0.2993^**^
		(−2.13)	(−2.13)	(−2.21)
2.employ		−0.3559^**^	−0.3564^**^	−0.3687^**^
		(−2.44)	(−2.44)	(−2.53)
lnincome		−0.0042	−0.0042	−0.0039
		(−0.78)	(−0.78)	(−0.72)
health		0.0357	0.0356	0.0358
		(1.46)	(1.46)	(1.47)
edu		0.0111	0.0109	0.0102
		(0.15)	(0.15)	(0.14)
lnLGCSM_Exp			0.0317	−0.1844
			(0.18)	(−0.96)
lnUrbanRoad_PerCapita				−0.5789
				(−1.28)
GreenRate				0.0606^***^
				(2.68)
Constant	0.7903^**^	1.1430	0.9833	0.1370
	(2.09)	(0.42)	(0.34)	(0.04)
Observations	23,957	23,957	23,957	23,957
*R*-squared	0.017	0.022	0.022	0.023
Number of pid	19,425	19,425	19,425	19,425
Individual FE	Yes	Yes	Yes	Yes
Province FE	Yes	Yes	Yes	Yes
Year FE	Yes	Yes	Yes	Yes

T statistics in parentheses. ^***^ and ^**^ indicate significance at the 1% and 5% levels, respectively.

The results for control variables show a significant relationship between marital status and exercise frequency. Compared to single individuals, married, and cohabiting individuals have notably lower exercise frequencies, consistent with previous studies (97–99). This suggests that marriage or partnership may suppress fitness behavior, likely due to increased family responsibilities and reduced leisure time. Employment status also shows a significant relationship with exercise frequency, aligning with existing research (100). Employed individuals have significantly lower sports participation compared to the unemployed, possibly due to work-related stress and time constraints. Similarly, individuals who have exited the labor force also experience a significant decline in exercise frequency. This may indicate that their fitness behavior does not improve post-retirement, and instead, factors such as poor time allocation or lack of motivation contribute to reduced exercise frequency. Green coverage has a positive and statistically significant effect on exercise frequency at the 1% level (0.0606), in line with previous studies (101). Improvements in environmental greening significantly promote fitness behavior among middle-aged and young individuals. An increase in green coverage may enhance the accessibility and comfort of fitness locations, thereby boosting individuals' motivation to exercise.

### 4.2 Endogeneity test

Economic variables often exhibit varying degrees of endogeneity, and severe endogeneity can compromise the unbiasedness and consistency of estimation results. In this study, the endogeneity issue primarily arises from the potential reverse causality between climate risk and individual sports participation. On the one hand, climate risk significantly influences individuals' fitness behaviors; on the other hand, individuals who engage in green, low-carbon exercise may reduce environmental pollution and contribute to climate governance (102). Additionally, fitness enthusiasts may be more aware of environmental pollution, leading them to advocate for or participate in environmental protection efforts, which could reduce the occurrence of extreme climate events and subsequently affect the frequency of climate risk changes. To address potential endogeneity, the most common approach is to select appropriate instrumental variables and perform consistent estimation through two-stage least squares (2SLS). Drawing from the approach of Chen and Zhang (14), this study selects the average climate risk of other provinces in the same year as an instrumental variable. This instrument is theoretically more exogenous, as the climate risk of other provinces is not directly influenced by the behavior of individual subjects and effectively captures cross-regional climate fluctuations.

After applying the two-stage least squares (2SLS) method, the first column of Table 4 presents the first-stage regression results of the instrumental variable approach, while the second column reports the second-stage regression outcomes. The regression results show that the Anderson Canonical Correlation LM statistic is significant, and the Cragg-Donald Wald F statistic is greater than the critical value of the Stock-Yogo *F*-test. This allows the rejection of the null hypotheses of “insufficient instrument variable identification” and “weak instrument variable identification,” thereby confirming the validity of the selected instrument variables. Furthermore, the results in the second column of Table 4 show that the coefficient for the climate risk index is negative and statistically significant at the 5% level, suggesting that, after accounting for endogeneity, climate risk continues to exert a significant negative effect on individual sports participation. This strengthens the robustness of the regression findings.

**Table 4:** Two-stage least squares test.

**Variables**	**(1)**	**(2)**
	**CPRI**	**ef**
CPRI_iv	0.9948^***^	
	(65.74)	
CPRI		−0.0106^**^
		(−2.53)
age		0.0035
		(0.05)
**Marital status (with single as the reference category)**		
2.marital		−0.3158^***^
		(−2.86)
3.marital		0.0491
		(0.22)
**Employment status** ***(with unemployed as the***		
***reference category)***		
1.employ		−0.2989^**^
		(−2.21)
2.employ		−0.3684^**^
		(−2.54)
lnincome		−0.0039
		(−0.72)
health		0.0359
		(1.48)
edu		0.0106
		(0.14)
lnLGCSM_Exp		−0.1871
		(−0.98)
lnUrbanRoad_PerCapita		−0.5645
		(−1.25)
GreenRate		0.0602^***^
		(2.68)
Constant	−0.2067	
	(−0.31)	
Observations	23,957	8,685
*R*-squared	0.488	0.023
Number of pid	19,425	4,153
Individual FE	No	Yes
Province FE	No	Yes
Year FE	No	Yes
Anderson canon. corr. LM statistic	4,518.198^***^
Cragg-Donald Wald F statistic	1.5e+06

^***^ and ^**^ indicate significance at the 1% and 5% levels, respectively.

### 4.3 Robustness test

#### 4.3.1 Substitute the dependent variable

First, to test the robustness of the impact of climate risk on sports participation, this study uses the log-transformed exercise time (lnet) as an alternative indicator. This variable is based on the exercise times (in minutes) from the CFPS data, with 1 added to the values before taking the logarithm to improve the data distribution and mitigate the impact of extreme values. The regression results in the first column of Table 5 show that the coefficient of the CPRI is −0.0129 and significant at the 5% level, indicating that an increase in climate risk significantly reduces the exercise time of middle-aged and young individuals. This further confirms the robustness of the findings.

**Table 5:** Robustness test: substitution of explanatory and dependent variables.

**Variables**	**(1)**	**(2)**	**(3)**
	**lnet**	**ef_dummy**	**ef_dummy**
CPRI	−0.0129^**^	−0.0027^**^	−0.0170^**^
	(−2.33)	(−2.11)	(−2.13)
age	−0.0590	−0.0133	−0.0685
	(−0.67)	(−0.66)	(−0.65)
**Marital status (with single as the reference category)**			
2.marital	−0.5073^***^	−0.1143^***^	−0.6275^***^
	(−3.47)	(−3.40)	(−3.21)
3.marital	−0.2886	−0.0811	−0.3749
	(−0.99)	(−1.21)	(−1.00)
**Employment status (with unemployed as the**			
**reference category)**			
1.employ	−0.3388^*^	−0.1127^***^	−0.7352^***^
	(−1.89)	(−2.73)	(−2.68)
2.employ	−0.4584^**^	−0.1347^***^	−0.8854^***^
	(−2.38)	(−3.04)	(−3.06)
lnincome	−0.0056	−0.0007	−0.0044
	(−0.78)	(−0.45)	(−0.45)
health	0.0157	0.0048	0.0322
	(0.49)	(0.65)	(0.72)
edu	−0.0431	−0.0105	−0.0658
	(−0.44)	(−0.46)	(−0.51)
lnLGCSM_Exp	−0.2358	−0.0642	−0.4167
	(−0.93)	(−1.10)	(−1.20)
lnUrbanRoad_PerCapita	−0.5170	−0.1031	−0.1444
	(−0.87)	(−0.75)	(−0.17)
GreenRate	0.0740^**^	0.0165^**^	0.1022^**^
	(2.49)	(2.41)	(2.41)
Constant	3.3653	0.8138	
	(0.83)	(0.87)	
Observations	23,957	23,957	3,176
*R*-squared	0.022	0.022	
Number of pid	19,425	19,425	1,484
Individual FE	Yes	Yes	Yes
Province FE	Yes	Yes	Yes
Year FE	Yes	Yes	Yes

T statistics in parentheses. ^***^, ^**^, ^*^ indicate significance at the 1%, 5%, 10% levels, respectively.

Second, drawing on the methodology of Guanglai and Ning (103), this study labels samples with a response value of 0 for both exercise frequency and duration as “non-participants in physical exercise,” and samples with non-zero responses as “participants in physical exercise.” This results in the construction of a binary variable, exercise participation (ef_dummy), to measure whether an individual engages in physical exercise. The regression results in the second column of Table 5 show that the coefficient of the climate risk index is −0.0027 and significant at the 5% level, indicating that an increase in climate risk significantly reduces the likelihood of exercise participation. Furthermore, since ef_dummy is a binary variable, the study also employs a logistic regression model to further test the robustness of the results, as shown in the third column of Table 5. The coefficient of the climate risk index is −0.0170 and remains significantly negative at the 5% level. This finding confirms that the negative impact of climate risk on exercise participation remains robust even when analyzed using a non-linear model. Additionally, the direction and significance of variables such as marital status, employment status, and greenery coverage are consistent with the previous model results.

#### 4.3.2 Change of statistical model

Third, an alternative econometric model is applied. In the first column of Table 6, the standard errors of the model are further adjusted for robustness to control for potential estimation bias due to heteroscedasticity. The results show that the coefficient of the CPRI is −0.0100 and remains statistically significant at the 5% level. This indicates that even after adjusting for standard errors, climate risk significantly reduces the exercise participation of middle-aged and young individuals, confirming the robustness of the results.

**Table 6:** Robustness test: model modification.

**Variables**	**(1)**	**(2)**	**(3)**	**(4)**
	**ef**	**ef**	**ef**	**ef**
CPRI	−0.0100^**^	−0.0188^***^	−0.0087^**^	−0.0114^***^
	(−2.46)	(−4.54)	(−2.01)	(−2.59)
age	0.0036	0.0325^***^	0.0038	−0.0338
	(0.06)	(12.70)	(0.06)	(−0.49)
**Marital status** ***(*****with single as the reference category)**				
2.marital	−0.3161^***^	−0.5117^***^	−0.3246^***^	−0.2639^**^
	(−2.71)	(−10.44)	(−2.92)	(−2.21)
3.marital	0.0482	−0.0669	0.0480	0.1816
	(0.18)	(−0.61)	(0.22)	(0.78)
**Employment status (with unemployed as the**				
**reference category)**				
1.employ	−0.2993^**^	−0.1914	−0.2943^**^	−0.2890^**^
	(−2.49)	(−1.64)	(−2.16)	(−2.05)
2.employ	−0.3687^***^	−0.1959	−0.3824^***^	−0.3777^**^
	(−2.79)	(−1.54)	(−2.59)	(−2.49)
lnincome	−0.0039	0.0049	−0.0027	−0.0040
	(−0.72)	(1.28)	(−0.45)	(−0.71)
health	0.0358	0.0924^***^	0.0361	0.0292
	(1.44)	(5.93)	(1.48)	(1.15)
edu	0.0102	0.3540^***^	0.0042	0.0071
	(0.12)	(24.25)	(0.06)	(0.09)
lnLGCSM_Exp	−0.1844	−0.2925	−0.1943	−0.3075
	(−0.92)	(−1.63)	(−0.99)	(−1.59)
lnUrbanRoad_PerCapita	−0.5789	−0.5673	−0.6510	−0.3718
	(−1.35)	(−1.44)	(−1.42)	(−0.96)
GreenRate	0.0606^***^	0.0467^**^	0.0413	0.0439^**^
	(2.72)	(1.98)	(1.50)	(1.97)
work_hour			−0.0094	
			(−0.45)	
bmi			0.0021	
			(0.64)	
lnRetailSales			0.2346	
			(1.25)	
lnBeds_Healthcare			−0.6044	
			(−0.49)	
Constant	0.1370		0.4536	3.8343
	(0.05)		(0.12)	(1.21)
Observations	23,957	23,957	23,957	23,953
R-squared	0.023		0.024	0.106
Number of pid	19,425	19,425	19,425	19,423
Individual FE	Yes	Yes	Yes	Yes
Province FE	Yes	Yes	Yes	No
County FE	No	No	No	Yes
Year FE	Yes	Yes	Yes	Yes

T statistics in parentheses. ^***^, ^**^, ^*^ indicate significance at the 1%, 5%, 10% levels, respectively.

Fourth, since the dependent variable (exercise frequency) is an ordered categorical variable, an ordered Logit model is used for analysis in the second column of Table 6. The ordered Logit model is better suited for handling ordered categorical data and effectively estimates the relative risks or probability changes between different categories. The results show that the coefficient of the climate risk index is −0.0188 and is statistically significant at the 1% level. This further indicates that climate risk significantly reduces the probability of individual exercise participation. Among the control variables, age, marital status, education level, and health status are significantly related to exercise frequency in this model. For example, compared to single individuals, married or cohabiting individuals are more likely to maintain a lower exercise frequency.

Fifth, five additional key control variables were included in Column 3 to more comprehensively reflect the personal characteristics and external environmental factors influencing fitness behavior. These variables were chosen because they provide a holistic view of the factors affecting fitness behavior. Work hours (work_hour) influence an individual's free time, directly determining the likelihood of exercising; body mass index (bmi) is closely related to health status and may affect an individual's motivation and ability to exercise; total retail sales of consumer goods (lnRetailSales) reflect economic activity and consumer spending power, influencing the availability of health resources and facilities in the region; the number of healthcare beds (lnBeds_Healthcare) represents regional medical resources and health security, thereby impacting residents' health behaviors; and local government spending on culture, sports, and media (lnLGCSM_Exp) reflects the government's investment in these areas, directly affecting the provision of exercise facilities and health programs. By controlling for these variables, external confounding factors can be better accounted for, allowing for a more accurate analysis of the determinants of fitness behavior. After introducing these variables, the coefficient of the climate risk index remains −0.0087 and is significant at the 5% level. This indicates that even after controlling for additional personal and external environmental factors, the negative impact of climate risk on fitness behavior persists, further validating the robustness of the results.

Sixth, to further verify the robustness of the results, we used county-level fixed effects in Column 4 of Table 6 for the analysis, rather than provincial-level fixed effects. County-level fixed effects allow for a more detailed control of regional heterogeneity, eliminating the impact of unobserved factors at the county level on individual fitness behavior. The results show that the coefficient of the climate risk index is −0.0114, which is statistically significant at the 1% level. This result is consistent with the main regression, further confirming the robustness of the negative impact of climate risk on exercise participation.

### 4.4 Long-term effect test

To examine the long-term impact of climate risk on individual exercise participation, this study constructs a regression model that includes lagged terms of the climate risk index. The model introduces lagged variables of the climate risk index for one period (l_CPRI), two periods (l2_CPRI), and three periods (l3_CPRI) to assess their effects on individual exercise participation (ef). The model is as follows:


(2) $$\begin{array}{l} ef_{i,t}={{\beta_{0}+\beta }_{1}CPRI}_{i,t-1}/\frac{CPRI_{i,t-2}}{CPRI_{i,t-3}}+\beta_{2}Z_{i,t}+\delta_{t}+\varphi_{i}+\varepsilon_{i,t} \end{array}$$

In the model, in addition to controlling for potential influencing factors such as age (age), marital status (marital), employment status (employ), income level (lnincome), health status (health), education level (edu), lifestyle (lnLGCSM_Exp), urban infrastructure (lnUrbanRoad_PerCapita), and green space coverage rate (GreenRate), the model also accounts for fixed effects of provinces and years to eliminate systematic regional and temporal differences.

Table 7 presents the long-term impact of the CPRI on individual exercise participation. The results indicate that climate risk has varying effects on exercise participation across different time scales. Specifically, the lagged variable for the first period of climate risk is not significant, while the second and third-period lagged variables both show a significant positive impact on exercise participation, suggesting that over time, climate risk promotes participation in physical activities. This result implies that, over a longer time frame, an increase in climate risk may encourage individuals to engage more in exercise. In the long run, environmental uncertainty caused by climate change may drive individuals to place greater emphasis on health and fitness, thereby increasing participation in physical activities.

**Table 7:** Long–term test.

**Variables**	**(1)**	**(2)**	**(3)**
	**ef**	**ef**	**ef**
l_CPRI	−0.0032		
	(−0.68)		
l2_CPRI		0.0141^***^	
		(3.01)	
l3_CPRI			0.0114^**^
			(2.14)
age	0.0038	0.0036	0.0010
	(0.06)	(0.05)	(0.01)
**Marital status (with single as the reference category)**			
2.marital	−0.3204^***^	−0.3248^***^	−0.3215^***^
	(−2.89)	(−2.93)	(−2.90)
3.marital	0.0340	0.0398	0.0509
	(0.15)	(0.18)	(0.23)
**Employment status (with unemployed as the**			
**reference category)**			
1.employ	−0.3056^**^	−0.3059^**^	−0.3017^**^
	(−2.25)	(−2.26)	(−2.22)
2.employ	−0.3730^**^	−0.3737^**^	−0.3760^***^
	(−2.56)	(−2.56)	(−2.58)
lnincome	−0.0040	−0.0044	−0.0041
	(−0.74)	(−0.82)	(−0.77)
health	0.0353	0.0343	0.0346
	(1.44)	(1.41)	(1.41)
edu	0.0039	0.0054	0.0056
	(0.05)	(0.07)	(0.07)
lnLGCSM_Exp	−0.1102	−0.3526^*^	−0.1895
	(−0.56)	(−1.73)	(−0.98)
lnUrbanRoad_PerCapita	−0.8394^*^	−0.9747^**^	−0.6209
	(−1.90)	(−2.20)	(−1.38)
GreenRate	0.0673^***^	0.0640^***^	0.0688^***^
	(3.00)	(2.85)	(3.07)
Constant	−0.4180	1.3369	−0.3487
	(−0.14)	(0.43)	(−0.11)
Observations	23,957	23,957	23,957
R–squared	0.022	0.024	0.023
Number of pid	19,425	19,425	19,425
Individual FE	Yes	Yes	Yes
Province FE	Yes	Yes	Yes
Year FE	Yes	Yes	Yes

T statistics in parentheses. ^***^, ^**^, ^*^ indicate significance at the 1%, 5%, 10% levels, respectively.

## 5 Mechanism analysis

The above analysis directly demonstrates the impact of climate risk on individual exercise participation. In this section, we will explore the underlying mechanisms. To examine the mediating roles of life satisfaction and digital lifestyle between climate risk and individual exercise participation, a four-stage mediation model is proposed for testing. This approach, by considering the relationship between the mediator and the dependent variable, enhances the completeness of the empirical framework. Based on the work of Zhiwei et al. (104) and following the design of Model (1), this study constructs the following mediation models (3), (4), and (5):


(3) $$\begin{array}{l} M_{i,t}={{\beta_{0}+\beta }_{1}CR}_{i,t-1}{{+\beta }_{2}Z}_{i,t-1}+\delta_{t}+\varphi_{i}+\varepsilon_{i,t}\; \end{array}$$

(4) $$\begin{array}{l} ef_{i,t}={{\beta_{0}+\beta }_{1}M}_{i,t-1}{{+\beta }_{2}Z}_{i,t-1}+\delta_{t}+\varphi_{i}+\;\varepsilon_{i,t}\; \end{array}$$

(5) $$\begin{array}{l} ef_{i,t}={{\beta_{0}+\beta }_{1}M}_{i,t-1}+{\beta_{2}CR}_{i,t-1}+{{+\beta }_{3}Z}_{i,t-1}+\delta_{t}+\varphi_{i}+\varepsilon_{i,t}\; \end{array}$$

The mediator variable (*M*_*i, t*_) is included, while also controlling for individual, time, and provincial fixed effects.

### 5.1 Life satisfaction

As discussed in the theoretical analysis, life satisfaction may play a significant mediating role between climate risk and individual exercise participation. When individuals face higher climate risks, factors such as psychological stress and environmental deterioration may lead to a decline in life satisfaction, thereby reducing their motivation and frequency of exercise participation. To test this hypothesis, life satisfaction (satisfy) is used as a mediator variable in the mediation analysis, with results presented in Table 8. The variable “satisfy” measures individual satisfaction. In the CFPS, if an individual answers “fairly satisfied” or “very satisfied,” the value of satisfy is 1, indicating high satisfaction; if the individual answers “very dissatisfied,” “fairly dissatisfied,” or “neutral,” the value of satisfy is 0, indicating low satisfaction.

**Table 8:** Channels through which climate risks effect sports participation: life satisfaction.

**Variables**	**(1)**	**(2)**	**(3)**
	**satisfy**	**ef**	**ef**
CPRI	−0.0024^*^		−0.0099^**^
	(−1.81)		(−2.34)
satisfy		0.0643	0.0612
		(1.35)	(1.28)
age	0.0164	0.0033	0.0025
	(0.79)	(0.05)	(0.04)
**Marital status (with single as the reference category)**			
2.marital	0.0636^*^	−0.3257^***^	−0.3200^***^
	(1.83)	(−2.94)	(−2.89)
3.marital	0.0611	0.0287	0.0444
	(0.88)	(0.13)	(0.20)
**Employment status (with unemployed as the**			
**reference category)**			
1.employ	0.0554	−0.3108^**^	−0.3027^**^
	(1.30)	(−2.29)	(−2.23)
2.employ	0.0434	−0.3761^***^	−0.3713^**^
	(0.95)	(−2.58)	(−2.55)
lnincome	0.0027	−0.0042	−0.0040
	(1.59)	(−0.77)	(−0.75)
health	0.0533^***^	0.0314	0.0325
	(6.97)	(1.28)	(1.33)
edu	0.0185	0.0021	0.0091
	(0.79)	(0.03)	(0.12)
lnLGCSM_Exp	0.0592	−0.1429	−0.1880
	(0.98)	(−0.75)	(−0.98)
lnUrbanRoad_PerCapita	0.1855	−0.8300^*^	−0.5903
	(1.31)	(−1.88)	(−1.30)
GreenRate	−0.0023	0.0671^***^	0.0607^***^
	(−0.33)	(2.99)	(2.69)
Constant	−0.9044	−0.2344	0.1924
	(−0.94)	(−0.08)	(0.06)
Sobel Z	−5.671^***^
Bootstrap (1,000 replicates) Testing Confidence Intervals	[−0.0007601, −0.000348]		
Observations	23,957	23,957	23,957
R–squared	0.069	0.022	0.024
Number of pid	19,425	19,425	19,425
Individual FE	Yes	Yes	Yes
Province FE	Yes	Yes	Yes
Year FE	Yes	Yes	Yes

T statistics in parentheses. ^***^, ^**^, ^*^ indicate significance at the 1%, 5%, 10% levels, respectively.

As shown in Table 8, in Column 1, the coefficient of CPRI is significantly negative, indicating that climate risk reduces individual life satisfaction. In Column 2, the coefficient between life satisfaction and sports participation is not significant, suggesting no direct linear relationship between the two. Therefore, this study further conducted the Sobel test, and the data results show a *Z*-value statistic of −5.671, significant at the 1% level. Additionally, the study employed Bootstrap sampling (1,000 iterations), revealing a 95% confidence interval for the mediation effect between (−0.0008 and −0.0004), which does not include 0. These results confirm that life satisfaction plays a mediating role. Specifically, climate risk reduces the life satisfaction of middle-aged and young individuals, which in turn suppresses their participation in physical exercise, thereby validating Hypothesis 1.

### 5.2 Digital lifestyles

Climate risk significantly affects individual participation in exercise, while the widespread adoption of modern digital technologies has provided more diverse options for engagement, with online fitness and digital courses becoming new directions for physical activity. Drawing on the research by Yupeng et al. (105), this study constructs an internet usage cognition variable (icon) based on the digital divide theory, which measures an individual's perception of the importance of internet usage, reflecting their level of digital awareness. The variable ranges from 1 (not important at all) to 5 (very important), capturing subjective cognitive differences across individuals. As shown in Table 9, in Column 1, the coefficient of CPRI is significantly positive, indicating that climate risk significantly enhances individuals' digital awareness. In Column 2, the coefficient between digital awareness and sports participation is significantly positive, suggesting a positive correlation between the two. Furthermore, in Column 3, the stepwise regression results show a positive coefficient for digital awareness, and the coefficient of CPRI is significant at the 5% level. These findings suggest that digital awareness plays a mediating role, meaning that climate risk increases individual' digital awareness, which in turn promotes participation in physical exercise, thereby validating Hypothesis 2.

**Table 9:** Channels through which climate risks effect sports participation: digital lifestyles.

**Variables**	**(1)**	**(2)**	**(3)**	**(4)**	**(5)**	**(6)**
	**icon**	**ef**	**ef**	**lnMobileUser**	**ef**	**ef**
CPRI	0.0073^**^		−0.0106^**^	0.0008^***^		−0.0109^***^
	(2.01)		(−2.52)	(3.87)		(−2.58)
icon		0.0753^***^	0.0766^***^			
		(4.36)	(4.44)			
lnMobileUser					1.0601^***^	1.1071^***^
					(3.36)	(3.50)
age	−0.0562	0.0087	0.0079	−0.0008	0.0053	0.0044
	(−0.98)	(0.13)	(0.12)	(−0.25)	(0.08)	(0.07)
**Marital status (with single as the reference category)**						
2.marital	−0.1373	−0.3117^***^	−0.3056^***^	0.0019	−0.3242^***^	−0.3182^***^
	(−1.44)	(−2.82)	(−2.76)	(0.36)	(−2.93)	(−2.88)
3.marital	0.0532	0.0275	0.0441			
	(0.28)	(0.12)	(0.20)			
**Employment status (with unemployed as the reference category)**						
1.employ	0.1648	−0.3203^**^	−0.3120^**^	0.0043	−0.3127^**^	−0.3041^**^
	(1.41)	(−2.36)	(−2.30)	(0.68)	(−2.31)	(−2.24)
2.employ	−0.1355	−0.3634^**^	−0.3583^**^	0.0059	−0.3800^***^	−0.3751^**^
	(−1.08)	(−2.50)	(−2.46)	(0.85)	(−2.61)	(−2.58)
lnincome	0.0068	−0.0045	−0.0044	0.0005^*^	−0.0045	−0.0044
	(1.47)	(−0.84)	(−0.82)	(1.81)	(−0.83)	(−0.81)
health	0.0077	0.0342	0.0352	0.0004	0.0343	0.0354
	(0.36)	(1.40)	(1.45)	(0.36)	(1.41)	(1.45)
edu	0.3407^***^	−0.0228	−0.0159	−0.0060^*^	0.0090	0.0169
	(5.28)	(−0.30)	(−0.21)	(−1.70)	(0.12)	(0.23)
lnLGCSM_Exp	−0.1141	−0.1272	−0.1756	−0.1671^***^	0.0425	0.0006
	(−0.69)	(−0.67)	(−0.91)	(−18.40)	(0.21)	(0.00)
lnUrbanRoad_PerCapita	−0.6327	−0.7875^*^	−0.5304	0.4352^***^	−1.3028^***^	−1.0607^**^
	(−1.62)	(−1.79)	(−1.17)	(20.37)	(−2.81)	(−2.24)
GreenRate	0.0339^*^	0.0649^***^	0.0580^**^	−0.0036^***^	0.0714^***^	0.0645^***^
	(1.74)	(2.90)	(2.57)	(−3.34)	(3.18)	(2.86)
Constant	2.0603	−0.4784	−0.0209	8.0769^***^	−8.8967^**^	−8.8049^**^
	(0.78)	(−0.16)	(−0.01)	(55.75)	(−2.23)	(−2.21)
Observations	23,957	23,957	23,957	23,957	23,957	23,957
R–squared	0.110	0.026	0.028	0.933	0.025	0.026
Number of pid	19,425	19,425	19,425	19,425	19,425	19,425
Individual FE	Yes	Yes	Yes	Yes	Yes	Yes
Province FE	Yes	Yes	Yes	Yes	Yes	Yes
Year FE	Yes	Yes	Yes	Yes	Yes	Yes

T statistics in parentheses. ^***^, ^**^, ^*^ indicate significance at the 1%, 5%, 10% levels, respectively.

At the same time, we further used the natural logarithm of the number of provincial mobile internet users plus one (lnMobileUser) as an alternative measure of digital lifestyle to assess its mediating role in the impact of climate risk on individual physical exercise. This indicator reflects the level of digital technology penetration at the regional level and the residents' dependence on digital services, providing a relatively objective measure of the popularity of individual digital lifestyles. The widespread use of mobile internet not only lowers the threshold for individuals to access fitness information and resources but also promotes diversified modes of sports participation, such as online fitness and the application of smart devices. Compared to individual-level digital awareness (icon), the number of mobile internet users can more directly reflect the level of digital infrastructure development and the overall societal acceptance of digital lifestyles. Therefore, using this indicator helps to further verify whether climate risk indirectly influences individual sports participation by promoting the widespread adoption of digital lifestyles. This provides a more concrete measure of how digital infrastructure and societal acceptance of digital services may mediate the relationship between climate risk and fitness behavior.

As shown in Table 9, in Column 4, the coefficient of CPRI is significantly positive, indicating that climate risk significantly increases the number of mobile internet users. This suggests that extreme climate events may encourage more people to rely on digital channels for socializing, consumption, and sports activities. In Column 5, the coefficient between the number of mobile internet users and sports participation is significantly positive, indicating that an increase in regional digitalization levels can promote individuals to engage more actively in physical exercise. Additionally, in Column 6, the stepwise regression results show that the coefficient of mobile internet users is positive, and the coefficient of CPRI is significant at the 1% level. These findings suggest that the digital lifestyle plays a mediating role, meaning that climate risk increases individuals' digital lifestyle levels, which in turn promotes sports participation. This further validates Hypothesis 2.

## 6 Further examination

The western region of China, as an economically underdeveloped area, faces significant disparities in infrastructure development and public service provision (such as fitness facilities and healthcare resources) compared to the eastern region. Additionally, the western region has a more complex climate, with a higher frequency of extreme weather events (e.g., droughts and floods), which may exacerbate the impact of climate risks on residents' daily lives. Given these environmental and economic constraints, residents in the western region may be more sensitive to climate change in their fitness behaviors. Compared to urban areas, rural regions also face limited resources for physical activity. Rural residents' participation in physical activities is constrained by factors such as the availability of spaces, facilities, and transportation, and due to engagement in outdoor agricultural work, extreme weather events may have more direct impacts on their work and daily lives. As a result, the frequency and modes of physical activity in rural areas may differ significantly from those in urban areas. Against this background, this study focuses on a heterogeneity analysis of the western and rural regions in this section, aiming to reveal the differential impacts of climate risks on exercise behaviors across different regions and household structures. This analysis not only enhances the understanding of the relationship between climate risks and fitness behaviors but also provides empirical evidence for developing more targeted climate adaptation and health promotion policies for different regions.

This study first examines regional heterogeneity by dividing the sample into two groups: the western region and the central/eastern regions. Column 1 of Table 10 shows that the coefficient of the CPRI for the western region sample is −0.0266, which is statistically significant at the 5% level. This indicates that climate risk has a significant negative impact on sports participation in the western region. Further analysis reveals that the lagged term of climate risk also exhibits a significant negative effect, especially the coefficient of the one-period lag (l_CPRI) at −0.0346, which is statistically significant at the 1% level. This suggests that the long-term impact of climate risk on the western region is also significant. In contrast, as shown in Column 2 of Table 10, the relationship between climate risk and sports participation in the central/eastern regions is not significant, implying that residents in these regions are not significantly hindered in their sports participation when facing climate risks. This may be due to the better fitness facilities, higher socioeconomic levels, and stronger climate adaptation capacity in the central/eastern regions.

**Table 10:** Heterogeneity test: western region.

**Variables**	**(1)**	**(2)**	**(3)**	**(4)**	**(5)**
	**Western Region**	**Central and Eastern Regions**	**Western Region**	**Western Region**	**Western Region**
	**ef**	**ef**	**ef**	**ef**	**ef**
CPRI	−0.0266^**^	−0.0076			
	(−2.56)	(−1.25)			
l_CPRI			−0.0346^***^		
			(−2.73)		
l2_CPRI				−0.0169	
				(−0.67)	
l3_CPRI					0.0020
					(0.13)
age	0.0871	−0.0265	0.0869	0.0770	0.0769
	(0.68)	(−0.34)	(0.68)	(0.60)	(0.60)
**Marital status (with single as the reference category)**					
2.marital	−0.4191^*^	−0.2872^**^	−0.4194^*^	−0.4658^**^	−0.4572^**^
	(−1.88)	(−2.22)	(−1.88)	(−2.08)	(−2.05)
3.marital	−0.7661	0.2559	−0.7905	−0.8122^*^	−0.8166^*^
	(−1.56)	(1.01)	(−1.61)	(−1.65)	(−1.66)
**Employment status (with unemployed as the reference category)**					
1.employ	−0.0409	−0.3713^**^	−0.0441	−0.0225	−0.0291
	(−0.14)	(−2.39)	(−0.15)	(−0.08)	(−0.10)
2.employ	−0.0156	−0.4921^***^	−0.0108	0.0306	0.0227
	(−0.05)	(−2.94)	(−0.03)	(0.10)	(0.07)
lnincome	0.0060	−0.0072	0.0054	0.0065	0.0065
	(0.57)	(−1.14)	(0.52)	(0.62)	(0.62)
health	0.0465	0.0265	0.0487	0.0435	0.0447
	(0.96)	(0.93)	(1.00)	(0.89)	(0.92)
edu	−0.0439	0.0404	−0.0325	−0.0686	−0.0674
	(−0.32)	(0.45)	(−0.23)	(−0.49)	(−0.48)
lnLGCSM_Exp	1.0147	−0.1724	0.3765	−0.4279	−0.2109
	(1.40)	(−0.76)	(0.66)	(−0.72)	(−0.34)
lnUrbanRoad_PerCapita	−1.1704	−0.0116	−2.0234^**^	−2.3821^***^	−2.5525^***^
	(−1.19)	(−0.02)	(−2.40)	(−2.76)	(−3.09)
GreenRate	0.0884^*^	0.0587^**^	0.0919^**^	0.0740	0.0810
	(1.89)	(2.13)	(1.96)	(1.58)	(1.46)
Constant	−5.7226	−0.0423	−0.2140	4.8819	3.5140
	(−0.75)	(−0.01)	(−0.03)	(0.71)	(0.47)
Observations	6,739	17,218	6,739	6,739	6,739
R–squared	0.043	0.024	0.044	0.038	0.037
Number of pid	5,644	13,855	5,644	5,644	5,644
Individual FE	Yes	Yes	Yes	Yes	Yes
Province FE	Yes	Yes	Yes	Yes	Yes
Year FE	Yes	Yes	Yes	Yes	Yes

T statistics in parentheses. ^***^, ^**^, ^*^ indicate significance at the 1%, 5%, 10% levels, respectively.

Further heterogeneity analysis of urban and rural residents reveals significant differences, as shown in the results of Table 11. Column 1 of Table 11 indicates that in the rural sample, the coefficient of the CPRI is −0.0032, which is not statistically significant. This suggests that climate risk does not have a significant direct inhibitory effect on sports participation in rural areas in the short term. Additionally, the lagged variables (l_CPRI, l2_CPRI, and l3_CPRI) in models 3–5 also show no significant impact. In contrast, the results in Column 2 of Table 11 indicate that in the urban sample, the coefficient of CPRI is −0.0124, which is statistically significant at the 10% level. This suggests that climate risk has a negative impact on sports participation in urban areas, though the effect is less significant compared to the strong negative correlation in the western region. Compared to rural areas, urban areas have better fitness facilities and coping capabilities, which may mitigate the negative effects of climate risk.

**Table 11:** Heterogeneity test: rural areas.

**Variables**	**(1)**	**(2)**	**(3)**	**(4)**	**(5)**
	**Rural Areas**	**Urban Areas**	**Rural Areas**	**Rural Areas**	**Rural Areas**
	**ef**	**ef**	**ef**	**ef**	**ef**
CPRI	−0.0032	−0.0124^*^			
	(−0.49)	(−1.96)			
l_CPRI			−0.0044		
			(−0.61)		
l2_CPRI				−0.0025	
				(−0.34)	
l3_CPRI					−0.0135
					(−1.61)
age	0.0348	−0.0093	0.0335	0.0328	0.0326
	(0.28)	(−0.11)	(0.27)	(0.27)	(0.26)
**Marital status (with single as the reference category)**					
2.marital	−0.0818	−0.3336^**^	−0.0834	−0.0838	−0.0845
	(−0.45)	(−2.02)	(−0.46)	(−0.46)	(−0.46)
3.marital	0.4846	0.1885	0.4875	0.4806	0.4531
	(1.33)	(0.60)	(1.34)	(1.32)	(1.24)
**Employment status (with unemployed as the**					
**reference category)**					
1.employ	−0.1407	−0.3776^**^	−0.1387	−0.1431	−0.1571
	(−0.63)	(−2.01)	(−0.62)	(−0.64)	(−0.70)
2.employ	−0.3227	−0.3968^**^	−0.3213	−0.3240	−0.3256
	(−1.34)	(−1.96)	(−1.34)	(−1.35)	(−1.36)
lnincome	−0.0043	0.0012	−0.0044	−0.0043	−0.0039
	(−0.55)	(0.15)	(−0.56)	(−0.55)	(−0.50)
health	0.0125	0.0522	0.0123	0.0114	0.0130
	(0.35)	(1.40)	(0.34)	(0.32)	(0.36)
edu	−0.1160	0.0305	−0.1131	−0.1144	−0.1086
	(−0.90)	(0.28)	(−0.88)	(−0.89)	(−0.84)
lnLGCSM_Exp	0.1100	−0.4436^*^	0.1752	0.1632	0.1847
	(0.33)	(−1.68)	(0.51)	(0.46)	(0.55)
lnUrbanRoad_PerCapita	−1.9180^***^	0.3399	−2.0145^***^	−1.9809^***^	−2.3247^***^
	(−2.60)	(0.50)	(−2.87)	(−2.77)	(−3.21)
GreenRate	0.0783^**^	0.0907^***^	0.0805^**^	0.0811^**^	0.0774^**^
	(2.12)	(2.76)	(2.20)	(2.22)	(2.12)
Constant	−1.4918	−1.3207	−1.8368	−1.8189	−1.0995
	(−0.27)	(−0.31)	(−0.33)	(−0.32)	(−0.20)
Observations	11,685	12,272	11,685	11,685	11,685
R–squared	0.031	0.037	0.031	0.031	0.033
Number of pid	9,821	10,125	9,821	9,821	9,821
Individual FE	Yes	Yes	Yes	Yes	Yes
Province FE	Yes	Yes	Yes	Yes	Yes
Year FE	Yes	Yes	Yes	Yes	Yes

T statistics in parentheses. ^***^, ^**^, ^*^ indicate significance at the 1%, 5%, 10% levels, respectively.

## 7 Conclusions and recommendations

### 7.1 Conclusions

This study, based on data from the 2014–2022 China Family Panel Studies (CFPS), integrates adaptive behavior theory and life satisfaction theory to examine both the short-term and long-term effects of climate risk on the exercise frequency of middle-aged and young individuals, along with the underlying mechanisms. It provides both theoretical insights and empirical evidence for understanding how individuals respond to the impacts of climate change across various temporal and spatial dimensions. The main conclusions of the study are as follows.

Firstly, climate risk significantly inhibits the fitness behavior of middle-aged and young individuals in the short term. Regardless of the proxy variables, model specifications, or estimation methods used, the negative effect of the Climate Physical Risk Index (CPRI) on exercise frequency remains both significant and robust. As climate risk intensifies, extreme weather events and environmental changes disrupt individuals' outdoor exercise routines, leading to reductions in both the frequency and duration of physical activity. This finding underscores that climate risk has a broad inhibitory effect on sports participation in the short term.

Secondly, the mechanism analysis shows that climate risk indirectly affects fitness behavior by reducing life satisfaction and increasing digital awareness. In response to the impacts of climate change, a decline in life satisfaction weakens individuals' motivation to engage in physical activity. Moreover, lagged variable analysis reveals that while climate risk negatively influences sports participation in the short term, individuals gradually adapt to environmental changes over time, leading to shifts in their physical activity patterns. Notably, after a two-period lag, the effect of climate risk on exercise frequency becomes positive, reflecting the gradual development of long-term adaptive behaviors such as digital fitness and indoor exercise.

Finally, the heterogeneity analysis reveals regional and group-based differences in the effects of climate risk. In western and rural areas, the short-term negative impact of climate risk on exercise frequency is more pronounced, likely due to limited fitness resources and infrastructure, making residents' health behaviors more susceptible to environmental changes. In contrast, in the central, eastern, and urban regions, the negative impact of climate risk on sports participation is relatively smaller, possibly due to better fitness facilities and higher adaptive capacity among residents. Additionally, neither the western nor rural regions exhibited significant positive adaptive effects in the long term, highlighting regional disparities in the ability to respond to climate change.

### 7.2 Policy recommendations

Based on the research findings, the following policy recommendations are proposed to mitigate the adverse effects of climate risk on the fitness behavior of middle-aged and young individuals, and to foster the integrated development of health and environmental adaptation.

Firstly, improve fitness infrastructure and enhance residents' adaptive capacity. In western and rural areas, which are more vulnerable to climate risk, greater investment should be directed toward public fitness facilities, with an emphasis on optimizing the distribution and accessibility of exercise spaces. Specifically, in regions prone to extreme weather events, it is essential to establish indoor fitness centers and multifunctional activity hubs that can offer alternative exercise spaces, even under harsh climatic conditions. Additionally, investments in infrastructure such as transportation networks and green spaces should be prioritized to improve the overall living environment, thus minimizing the barriers to sports participation caused by climate risk.

Secondly, promote health and fitness awareness and foster the development of digital fitness. In addition to promoting universal sports participation, public awareness campaigns should focus on strengthening residents' health consciousness and their capacity to adapt to climate change. Encouraging the use of digital fitness solutions, such as online workout programs and smart fitness equipment, can help reduce reliance on outdoor conditions, particularly for middle-aged and young individuals. For urban residents, integrating fitness into smart city initiatives could further facilitate the digital transformation of fitness services, allowing for more flexible and accessible exercise options.

Thirdly, implement region-specific policies to promote coordinated regional development. Given the disparities in economic development, infrastructure, and climate adaptation capacity across regions, policy-making should be context-specific. In the western and rural areas, the priority should be improving basic public services and enhancing the capacity for health promotion and climate adaptation. In contrast, central, eastern, and urban areas can focus on advancing green, low-carbon urban development and promoting models that integrate both health and environmental sustainability. Tailored, region-specific policies help to bridge the gap in health and fitness resources, fostering more equitable development across regions.

Lastly, strengthen policy coordination to integrate health and environmental objectives. Policies governing health and environmental issues should complement one another, with enhanced interdepartmental cooperation. National fitness, climate adaptation, and public health policies should be integrated into local government development plans, facilitating cross-sector collaboration and the creation of a cohesive policy framework encompassing sports, health, environmental protection, and education. Such coordination improve policy implementation and contribute to the dual objectives of health promotion and effective climate risk management.

Although this study has elucidated the dynamic impact of climate risk on the behavior of middle-aged and young adults and its underlying mechanisms, there are still some limitations. First, the study focuses on the middle-aged group and overlooks the differences in adaptation abilities across various age groups. Different age groups may have significantly different responses and adaptation capacities when facing climate risks, yet this study has not explored these differences in depth. Second, although fixed-effects models were used to control for heterogeneity at the individual, year, and provincial levels, a limitation of this approach is that it cannot account for time-varying differences between individuals. Although the fixed-effects model effectively controls for unobserved time-invariant individual characteristics and time-varying factors, it may not fully capture the potential factors at the individual level that change over time. Finally, despite including several control variables in the model to reduce omitted variable bias, there are still some factors that could influence sports participation which were not fully incorporated into the model due to data limitations.

To address the limitations mentioned above, future research can be improved and expanded in the following ways. First, future studies could consider incorporating different age groups (such as children, adolescents, and older adult individuals) into the analytical framework to explore the differences in fitness behaviors and adaptation abilities to climate risk across various age groups. This will help provide a basis for developing more targeted policies for different groups. By considering age-specific responses and adaptation strategies, policymakers can better address the needs of diverse populations in the context of climate risks and physical activity. Secondly, considering the limitations of the fixed-effects model, future research could adopt more flexible models, such as structural dynamic models, to more comprehensively capture time-varying factors at the individual level. This improvement would help in gaining a deeper understanding of how time-related changes in individual characteristics influence fitness behaviors. Additionally, future studies could expand the data dimensions by incorporating more potential variables, particularly those related to individual health status and lifestyle factors, thus enhancing the explanatory power of the model. Furthermore, future research could integrate objective measurement data (e.g., wearable device data) to verify the accuracy of self-reported data, thereby reducing the impact of self-report biases. This approach would offer more reliable insights into the relationship between climate risk and fitness behaviors. Finally, to thoroughly analyze the long-term impact of climate risks on health behaviors, especially the cumulative effects of climate change on health behaviors and its intergenerational impacts, future research should extend the time span and conduct long-term tracking studies. By implementing these improvements, future research will provide a more solid theoretical foundation and empirical evidence for a comprehensive understanding of the mechanisms through which climate risks influence sports participation. This approach would allow for a deeper exploration of the enduring consequences of climate change on individuals' behaviors across different generations, offering valuable insights for policy and intervention strategies.

## Data Availability

Publicly available datasets were analyzed in this study. This data can be found here: https://www.isss.pku.edu.cn/cfps/en/.
